# The long pollen tube journey and in vitro pollen germination of *Phalaenopsis* orchids

**DOI:** 10.1007/s00497-016-0280-z

**Published:** 2016-03-25

**Authors:** Jhun-Chen Chen, Su-Chiung Fang

**Affiliations:** 1 Biotechnology Center in Southern Taiwan, Academia Sinica, Tainan, 741 Taiwan; 2 Agricultural Biotechnology Research Center, Academia Sinica, Taipei, 115 Taiwan

**Keywords:** *Phalaenopsis aphrodite*, Pollen tube, Reproductive development, Orchid, In vitro germination

## Abstract

*****Key message***:**

**Pollen biology in** ***P. aphrodite***.

**Abstract:**

Orchids have a distinct reproductive program. Pollination triggers ovule development and differentiation within flowers, and fertilization occurs days to months after pollination. It is unclear how pollen tubes travel through the developing ovaries during ovule development and when pollen tubes arrive at the mature embryo sac to achieve fertilization. Here, we report a robust staining protocol to image and record the timing of pollen germination, progressive growth of pollen tubes in ovaries, and arrival of pollen tubes at embryo sacs in *Phalaenopsis aphrodite*. The pollen germinated and pollen tubes entered the ovary 3 days after pollination. Pollen tubes continued to grow and filled the entire cavity of the ovary as the ovary elongated and ovules developed. Pollen tubes were found to enter the matured embryo sacs at approximately 60–65 days after pollination in an acropetal manner. Moreover, these temporal changes in developmental events such as growth of pollen tubes and fertilization were associated with expression of molecular markers. In addition, we developed an in vitro pollen germination protocol, which is valuable to enable studies on pollen tube guidance and tip growth regulation in *Phalaenopsis* orchids and possibly in other orchid species.

## Introduction

During the plant sexual cycle, pollen germinates on the stigma, and pollen tubes grow within the style and reach an embryo sac within a few hours to several days. Various modifications in reproductive systems have evolved in plants to coordinate pollination and fertilization and achieve successful reproduction. For example, pollen may travel as a single grain or as an aggregated entity. Pollen forms different types of aggregated entities called pollen dispersal units or PDUs (Pacini 1997). In angiosperms, Orchidaceae plants have the greatest number of PDU types and pollen is often packed in PDUs called pollinia (Pacini 2009; Pacini and Hesse 2002). The advantage of using PDU as a mode of fertilization is that it is highly effective with nearly all the ovules being fertilized and large amounts of seeds being produced by pollination.

In most angiosperms, the ovule structure and embryo sac have fully developed by the time pollen grains germinate. As a result, fertilization normally occurs hours after pollination (Christensen et al. 1997; Faure et al. 2002; Mòl et al. 1994; Wu et al. 2011). In other plant species such as orchids and Fagales, female gametophyte development is either absent or incomplete before pollination (Liu et al. 2014; O’Neill 1997; Pimienta and Polito 1983; Sogo and Tobe 2006a, b). For these plants, pollination is important to trigger or regulate embryo sac development and ovule maturation that subsequently allows fertilization. Orchid species provide notable examples of this kind of modified reproduction system (Arditti 1992; Yeung and Law 1997; Zhang and O’Neill 1993). For example, pollination triggers initiation and development of ovules that are absent in unpollinated ovaries of *Cattleya*, *Phalaenopsis,* and *Dendrobium* (Duncan and Curtis 1943; Israel and Sagawa 1964; Zhang and O’Neill 1993). In *Cypripedium* and *Paphiopedilum*, pollination triggers development of the ovule primordial that is present but arrested in the pre-meiotic stage before pollination (Duncan and Curtis 1942). It has been proposed that pollination-triggered female reproductive development ensures efficient investment in megagametophyte and ovary maturation for fertilization only after the low probability occurrence of pollination by highly specified pollinators (O’Neill 1997). In these cases, pollination not only provides paternal nuclei that contribute to the genetic makeup of the zygote, it also serves as a primary signal to coordinate developmental events required for successful fertilization (Zhang and O’Neill 1993).

Because of extended temporal separation between pollination and fertilization, germinated pollen and pollen tubes have to survive a significant period of time in the ovaries before entering the mature embryo sacs. It has been reported that this period of time can last as little as 4 days for *Gastrodia elata* to 10 months for *Vanda suavis* (Arditti 1992). However, the timely tracking of growth of the pollen tube between pollination and fertilization in orchid ovary remains limited. One reason for this is the lack of a reliable staining protocol to monitor pollen tubes in ovaries. Here, we report a robust pollen tube staining protocol that enables timely observation of the progression of pollen tube elongation in the developing ovaries of *Phalaenopsis aphrodite*. Using this protocol, we were able to pinpoint the timing when pollen tubes entered the matured ovaries. Pollen tubes are one of the best model systems for cellular process studies involved in polarity and tip growth (Krichevsky et al. 2007; Qin and Yang 2011; Yang 2008). To this end, we established an in vitro pollen germination and tube growth system, which will be valuable for studies of cell growth and morphogenesis of orchid pollen in the future.

## Materials and methods

### Plant materials and growth conditions

**Fig. 1:**
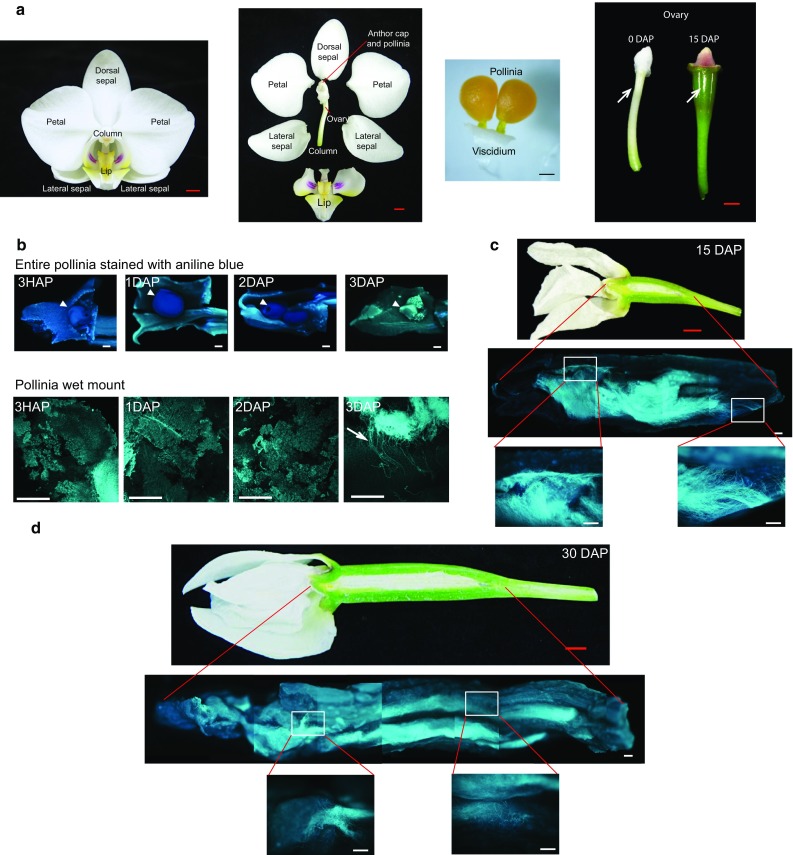
Aniline blue staining of pollinia and pollen. **a** Structure of a *P. aphrodite* flower (complete and dissected); pollinia attached to the viscidium; and ovaries before and after pollination. *White arrows* point to the ovaries at 0 and 15 days after pollination (DAP). Notice the enlargement of ovary at 15 DAP. **b** Images showing aniline blue staining of pollinia 3 h after pollination (HAP), 1, 2, and 3 days after pollination (DAP). *White arrowheads* point to the pollinia. *White arrow points* to the germinated pollen tubes. **c** Image showing developing capsule and distribution of pollen tubes in ovary collected at 15 days after pollination (DAP). Aniline blue staining images were assembled from separate images of the same ovary. High magnification images of the indicated areas are shown at the *bottom* of the assembled ovary. **d** Image showing developing capsule and distribution of pollen tubes in an ovary collected at 30 days after pollination (DAP). Aniline blue staining pictures were assembled from separate images of the same ovary. High magnification images of the indicated areas are shown at the *bottom* of the assembled ovary. *Black scale bar* 500 μm. *White scale bar* 500 μm. *Red scale bar* 0.5 cm

*Phalaenopsis aphrodite* subsp. *formosana* (m1663, tetraploid) seedlings in 2.5- or 3-inch pots were purchased from Chain Port Orchid Nursery (Ping Tung, Taiwan). Plants were grown in a growth chamber with alternating 12 h light (23 °C)/12 h dark (18 °C) cycles to induce flowering. The floral stalks (~0.5 to 1 cm long) became visible approximately 2 months after moving into the growth chamber. The first open flower appeared approximately three to 4 months after moving into the growth chamber. Flowers were hand pollinated 1 week after flower opening. Pollinia used for in vitro germination were taken from flowers from fully blooming flower spikes. For hand pollination, the pollinium was put in the stigmatic cavity. Following pollination, pollen grains adhered to the stigmatic cells. The stigma closed and then swelled to completely enclose the pollinium 1 day after pollination (DAP). The ovary started to develop and enlarge in size (Fig. 1a).

### Tissue fixation and pollen tube staining

Capsules, the developing ovaries, were cut open longitudinally and dissected transversely into approximately 3-cm-long segments. The capsule segments were fixed in 8:1:1 of 80 % ethanol, glacial acetic acid, and formalin solution at 4 °C overnight. The fixed samples were subsequently rehydrated by passing through 70, 50, and 30 % ethanol for 10 min and left in H_2_O at 4 °C overnight. Samples were cleared in 8 N NaOH at room temperature overnight, washed three times with H_2_O, and left in H_2_O at room temperature overnight. Aniline blue staining of germinated pollen tubes was performed as previously described (Martin 1959) with slight modifications. The capsule segments were stained with 0.1 % aniline blue in 0.1 M K_3_PO_4_ buffer and 2 % glycerol (v/v) in the dark overnight. The stained segments could be kept in the aniline blue staining solution at 4 °C for at least a couple of weeks without tissue deterioration.

### Microscopy imaging

LSM 710 confocal microscope (Zeiss) was use to score the arrival of pollen tubes at the micropyle end of ovules and to image pollen germination on the stigma. Aniline blue fluorescence was excited using the 405 nm laser line from a diode laser and the emitted light was filtered through a 410–471 band-pass filter. The callose staining pollen tubes were photographed under a 40 × C-Apochromat lens or a 100× Plan-Apochromat lens.

Distribution of aniline blue-stained pollen tubes in the developing capsules was photographed on a Zeiss Axio Scope A1 florescence microscope under a 2.5 × Plan-Neofluar lens or 5 × N-Achroplan lens with an AxioCam HRc camera (Zeiss).

### In vitro germination of pollen tubes

Stigma extract was prepared as previously described (Chen and Jiang 2014) with slight modification. Briefly, the stigma was removed and sterilized by 70 % EtOH for 20 s. Five removed stigma were vacuum drawn in 3 ml sterilized H_2_O to obtain soluble stigma extract. The stigma extract was filtered by 100-micron nylon mesh (Fisher Scientific, USA) and 100 μl of filtered stigma extract was added into 1 ml germination media described below. Pollinia were briefly sterilized by 0.05 % NaClO and rinsed with sterilized H_2_O. Pollinia were then squashed into small pieces before adding into the germination medium. Pollen tubes were allowed to germinate in modified BK medium containing 100 mg/l H_3_BO_3_, 100 mg/l CaCl_2_ · 2H_2_O, 100 mg/l MgSO_4_ · 7H_2_O, and 100 mg/l KNO_3_ supplemented with 10, 20, or 30 % sucrose, pH 5.7 (Tsai and Chang 2010), or in 10, 20, or 30 % sucrose solution.

For acetocarmine staining, fresh pollen and germinated pollen tubes were directly fixed and stained in acetocarmine solution containing 10 % acetic acid and 2 % carmine (Tokyo Chemicals Industry, Japan) on a microscope slide before visualization. To determine the growth rate of the pollen tube, the fixed pollen tubes were photographed 10, 20, or 30 day after incubation. The length of pollen grains and pollen tubes was measured by ImageJ software (Abràmoff et al. 2004). At least 50 pollen tubes were measured at each time point. Thirty-two pollen grains were measured to determine the average length of a pollen grain. The average length of a pollen grain is 17 μm.

### Scanning electron microscopy

Samples were fixed in 4 % paraformaldehyde and 2.5 % glutaraldehyde in 67 mM PBS buffer (pH 7.0). A vacuum was applied to remove air bubbles from tissues. Samples were incubated in the fixative at 4 °C overnight. After washing the samples with PBS buffer, they were incubated in 1 % OsO_4_ at room temperature for 4 h followed by washing with PBS buffer. Samples were then dehydrated in an ethanol series, critical point-dried with a critical point dryer (Hitachi HCP-2, Japan), sputter-coated with gold and platinum with Hitachi E-1010 ion sputter (Japan), and observed under a scanning electron microscope (FEI Quanta 200, USA) with an accelerating voltage of 20 kV.

### Sample collection and RNA extraction

Orchid flowers were hand pollinated, and developing ovaries were harvested on a specified day. For reproductive tissues, only the interior tissues of developing capsules were scooped and pooled for RNA extraction (Lin et al. 2014). Protocorm-like bodies (PLBs) and protocorms were collected. The samples were flash frozen in liquid nitrogen and stored in a freezer at −80 °C. For samples used in semi-quantitative RT-PCR, total RNA was isolated using OmicZol™ RNA Plus extraction reagent (Omics Bio) according to the manufacturer’s instructions. For samples used in quantitative RT-PCR, total RNA was isolated using TRIzol reagent (Invitrogen) followed by purification using Direct-Zol RNA MiniPrep kit (Zymo Research) according to the manufacturer’s instructions. The isolated total RNA was treated with RNase-free DNase (Qiagen) followed by RNeasy mini-column purification according to the manufacturer’s instructions (Qiagen).

### Quantitative and semi-quantitative RT-PCR

RNA was reverse transcribed in the presence of a mixture of oligo dT and random primers (9:1 ratio) using the GoScript Reverse Transcription System (Promega) as described previously (Lin et al. 2014). Ten microliters of quantitative RT-PCR reaction contained 2.5 μl of 1/20 diluted cDNA, 0.2 μM of primers, and 5 μl of 2 × KAPA SYBR FAST master mix (KAPA Biosystems). The following program was used for amplification: 95 °C for 1 min, 40 cycles of 95 °C for 5 s, and 58 or 60 °C for 20 s. PCR was performed in triplicate, and the experiments were repeated with RNA isolated from two independent samples. Primer pairs and the specified annealing temperature used for quantitative PCR are listed in Table 2. *Ubiquitin* (*PaUBI1*) was used as an internal control (Lin et al. 2014).

For semi-quantitative RT-PCR, twenty microliters of PCR reaction contained 1 μl of 1/2 diluted cDNA, 0.5 μM of primers, 80 μM dNTP, 3 % DMSO, and 0.2 μl of Power Taq DNA polymerase (Genomics BioSci & Tech, Taiwan). The following program was used for amplification: 95 °C for 2 min, 36 cycles of 94 °C for 25 s, 60 °C for 25 s, and 72 °C for 40 s. Primer pairs and the specified annealing temperature used for semi-quantitative PCR are listed in Table 2. The Genbank accession numbers of *PaC13L*, *PaLIM1*, and *PaEG1L1* are KU213915, KU213916, and KU213917, respectively (Table 1).

**Table 1:** Frequency of embryo sacs containing the pollen tubes at 50, 60, and 65 days after pollination

	No. of embryo sacs with pollen tubes			No. of embryo sacs without pollen tubes			Total no. of examined embryo sacs	% of embryo sacs with pollen tubes
	Up	M	Low	Up	M	Low		
50 DAP	0	0	3	33	32	37	105	3.0
60 DAP	8	14	31	59	30	31	173	30.6
65 DAP	31	12	13	33	22	4	115	48.7

*Up* upper parts of ovaries, *M* middle parts of ovaries, *Low* lower parts of ovaries. At least two capsules were used

**Table 2:** List of primer pairs used for RT-PCR and qRT-PCR

Gene name	Forward primer	Reverse primer	Amplicon size (bp)	Annealing temp (°C)
*PaC13L*	5′-CGCTGAGTTCGTTGAGAACA-3′	5′-GGCATACTGGAAGTGCAACA-3′	125	58
*PaLIM1*	5′-AACAGAGCAGCAAGCAAGGT-3′	5′-CGTAGCTCGATGGTGTGAGA-3′	172	60
*PaEC1L1*	5′-GCCATAACTGCTTCCTGCAT-3′	5′-GTAGCTCAATCAGCGCTTCC-3′	113	60
*PaUBI1*	5′-AACTCCATCGCCTTCCTCTT-3′	5′-TGAAGCATGGCATCAATTTC-3′	101	58, 60
*PaC13L*	5′-CAATTCAGGAGAGGCAAAGG-3′	5′-CCCCATGAATTGTTCCTTGA-3′	618	60
*PaLIM1*	5′-TTCTGCTCCGCTTTTGAAAT-3′	5′-ATATTATTGATCTTGTGTGTGTTGG-3′	647	60

## Results

### Pollen germination and progressive growth of the pollen tube during pollination-induced ovary development in *P. aprhodite*

For most angiosperms, ovules and embryo sacs have reached maturity by the time of pollination. Ovule development of orchids on the other hand is pollination-dependent. The progressive development of ovule and embryo structures after pollination in *Phalaenopsis* orchids has been reported (Chen et al. 2012; Tsai et al. 2008). However, the coordinated pollen germination and developmental processes are relatively limited. To monitor the growth of the pollen tube, aniline blue staining followed by confocal microscope imaging was used to visualize progressive growth of the pollen tube within the developing ovaries. Callose and the callose plug, commonly found in pollen grains and pollen tubes, can be stained selectively by water soluble aniline blue (Currier 1957). *Phalaenopsis aphrodite* was chosen as the system for our study because it is an important parental plant for commercial breeding programs in Taiwan and its pollination event has not been documented in detail.

*Phalaenopsis aphrodite* has small white-colored flowers. Each flower has two petals, one dorsal sepal, and two lateral sepals. The median petal is enlarged and modified to become a lip or labellum. The male and female reproductive parts are fused together and become the gynostemium or column (Fig. 1a). Pollen grains of *Phalaenopsis* orchids are packed together as a pollinium. Two pollinia were connected together and attached to a sticky viscidium, a disk-like structure that sticks to visiting insects. Pollinia sit on the top of the gynostemium under the anther cap (Fig. 1a).

**Fig. 2:**
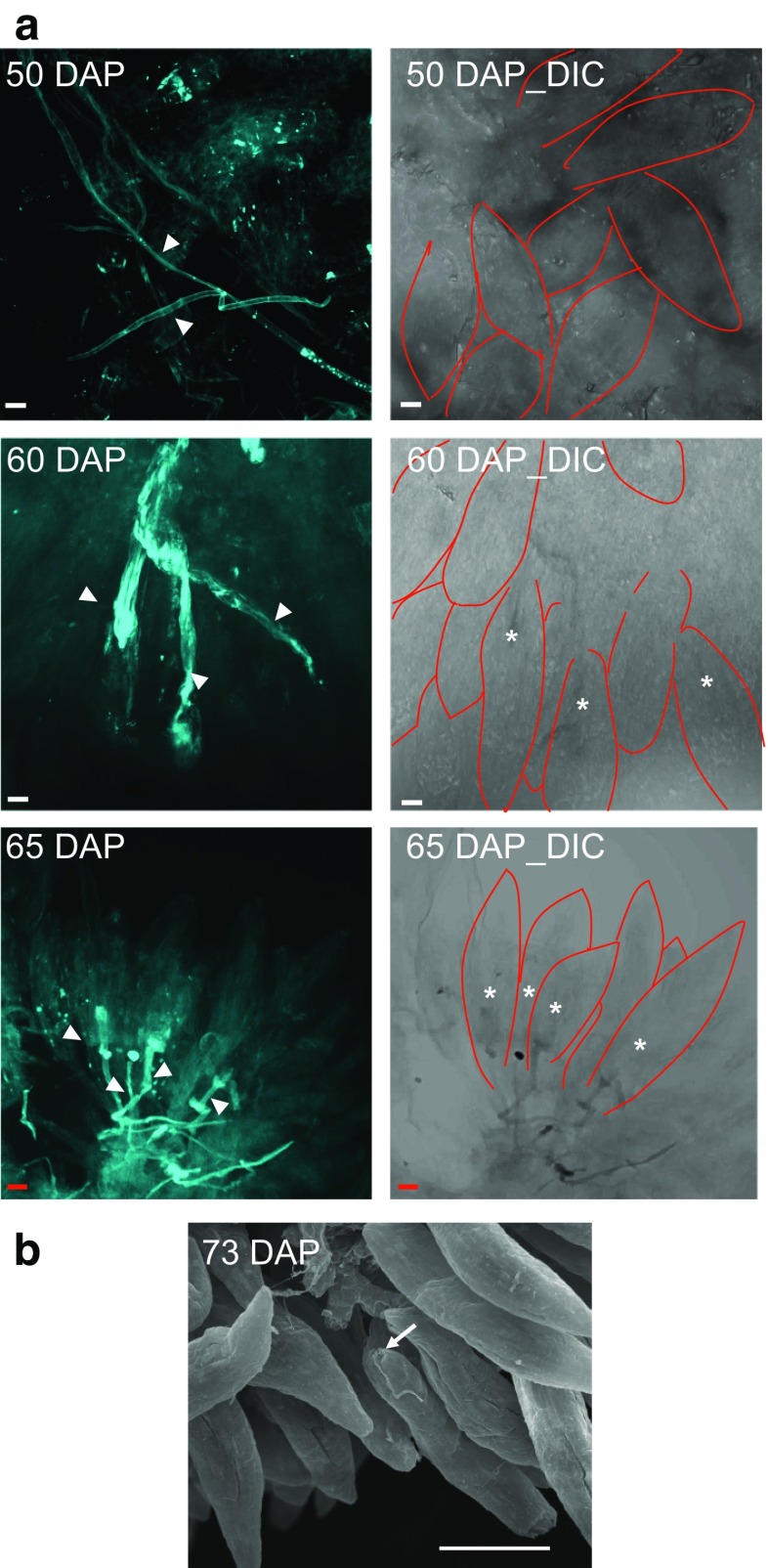
**a** Aniline blue staining images showing pollen tubes and developing ovules at 50, 65, and 70 days after pollination (DAP). The ovules under differential interference contrast (DIC) are outlined by fine *red lines* for better visualization. The *white asterisk* indicates the entry of the pollen tube into the embryo sac. The *white arrowheads* indicate the stained pollen tube. *White scale bar* 10 μm. *Red scale bar* 20 μm. **b** Scanning electron microscope image showing the entry of the pollen tube to the micropyle end of the matured female gametophyte at day 73 after pollination (73 DAP). *White arrow* indicates the micropyle end. *White scale bar* 100 μm

Following hand pollination, the pollen germinated and pollen tubes started to enter the ovary 3 days after pollination (Fig. 1b), which is earlier than previously reported (Zhang and O’Neill 1993). Fifteen days after germination, pollen tubes appeared to be evenly distributed in the cavities of both sides of the placenta and continued to grow as ovaries enlarged (Fig. 1c). The pollen tube continued to grow and distributed over the entire ovary cavities as the ovary grew at 30 DAP (Fig. 1d). To gain information about when pollen tubes were attracted to and reached the micropyle ends of the ovules to complete fertilization, aniline blue was used to track pollen tubes at 50, 60, 65, and 70 DAP. Only very few pollen tubes grew toward the micropyle ends of the ovules at 50 DAP (Table 1). The tips of pollen tubes started to enter the embryo sacs (30.6 %) at approximately 60 DAP, and the frequency reached up to 48.7 % at 65 DAP (Table 1; Fig. 2a). Intriguingly, fertilization events seemed to occur in an acropetal manner with micropyle-guided pollen tubes starting at the basal half (60 DAP) and gradually expanded to the upper half (65 DAP) of the developing ovaries (Table 1). Only a few pollen tubes showed aniline blue staining at 70 DAP, the point when fertilization took place. The presence of pollen tubes at the micropyle end of embryo sacs, despite its lack of aniline blue staining, at 73 DAP was confirmed by scanning electron microscopy analysis (Fig. 2b). Taken together, our data provide evidence that most fertilization events of *P. aphrodite* occurred between 65 DAP and 70 DAP.

### In vitro pollen germination system

**Fig. 3:**
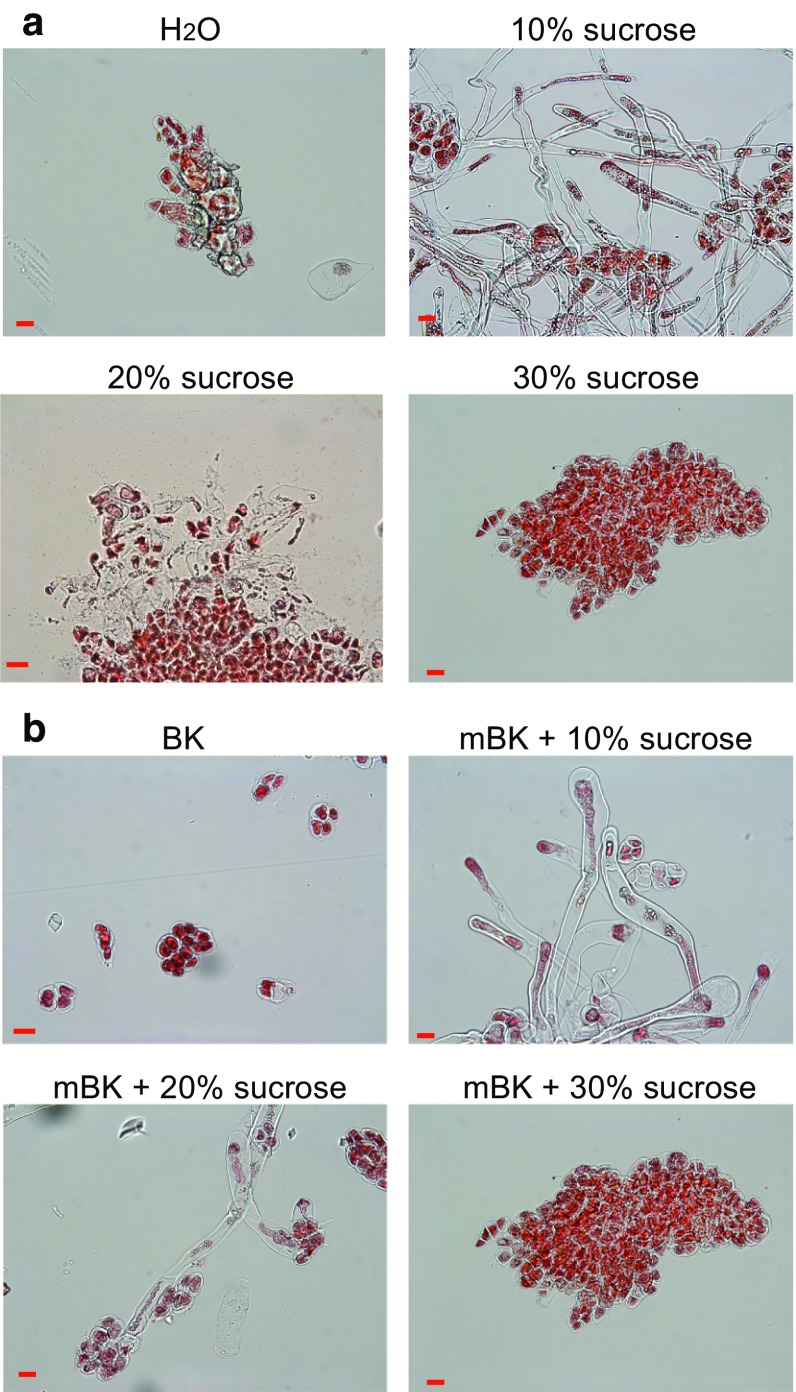
Pollen germinated 60 days after treatment under various conditions. **a** Pollen germinated in 0, 10, 20, and 30 % sucrose. **b** Pollen germinated in modified BK medium supplemented with 0, 10, 20, or 30 % sucrose. *Scale bar* 20 μm. Stigma tissue extract was added under all of the described conditions

The relatively long journey [from pollen germination to fertilization (Arditti 1992)] that orchid pollen tubes have to undergo within ovaries before reaching the embryo sacs to complete fertilization suggests that orchid pollen grains likely undergo unique development and differentiation processes. Establishment of an in vitro pollen germination and growth protocol provides a powerful tool to study pollen tube guidance and tip growth regulation (Boavida and McCormick 2007; Rodriguez-Enriquez et al. 2013) and is therefore an important tool to gain new insight into orchid pollen biology. To this end, the dissected pollinia of *P. aphrodite* flowers were allowed to germinate in a modified Brewbaker and Kwack (mBK) medium (Tsai and Chang 2010) or sucrose solution (Boavida and McCormick 2007). Germination is defined as the germinated tube growing to at least twice the length of a pollen grain. However, under the tested germination conditions, pollen grains failed to germinate. Because stigma extract has been shown to enhance pollen germination efficiency (Allen and Hiscock 2010; Roberts et al. 1983), stigma tissue extract was then added into the mBK medium or sucrose solution for pollen germination. In the presence of stigma tissue extract, pollen was able to germinate successfully in 10 and 20 % sucrose solution (Fig. 3a). In the presence of 10 % sucrose, pollen tubes grew steadily and continued to elongate 60 days after incubation. Pollen tubes germinated in 20 % sucrose on the other hand failed to elongate and appeared swollen 60 days after incubation. Some of the swollen pollen tubes eventually ruptured and broke into pieces. In the presence of stigma tissue extract, pollen grains were also able to germinate in mBK medium supplemented with either 10 or 20 % sucrose (Fig. 3b). With 10 % sucrose-supplemented mBK medium, pollen tubes elongated at a moderate rate but not as rigorously as with 10 % sucrose. With 20 % sucrose-supplemented mBK medium, some of the germinated pollen tubes became swollen and failed to elongate. Pollen tubes failed to germinate in the rest of the tested stigma extract supplemented media including H_2_O, 30 % sucrose, mBK medium, and mBK medium with 30 % sucrose (Fig. 3a, b). In summary, our data showed that 10 % sucrose provided the optimal condition and stigma extract is required for in vitro pollen germination of *P.* *aphrodite*.

**Fig. 4:**
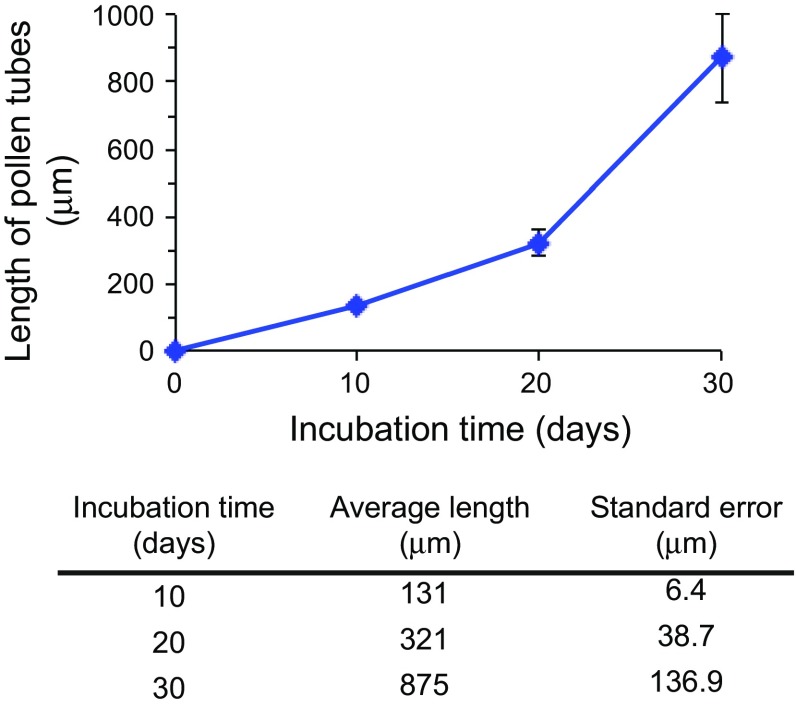
Length of in vitro germinated pollen tubes over time. At least 50 pollen tubes were measured and averaged after 10, 20, or 30 days of incubation

We next examined the growth rate of the tip-growing pollen tube by measuring lengths of pollen tubes in 10 % sucrose over time. Germination of pollen tubes was not synchronized. Typically, it took approximately 5–10 days to see the germinated pollen tubes (defined as twice the length of a pollen grain). Some pollen grains did not germinate until 10–20 days after incubation. Because it is difficult to separate long and tangled pollen tubes that remained attached to the dissected pollinia for measurement, the growth rate was not measured after 30 days of incubation. The pollen tubes elongated slowly initially (from day 10 to day 20) and grew at a relatively fast rate from day 20 to day 30 (Fig. 4). There was large variation in length of pollen tube after 30 days of incubation. It is likely caused by unsynchronized germination of pollen grains and/or uneven elongation rate of individual pollen tubes.

### Expression of pollen-specific and egg-specific genes is associated with growing pollen tubes and the timing of fertilization in *P. aprhodite*

**Fig. 5:**
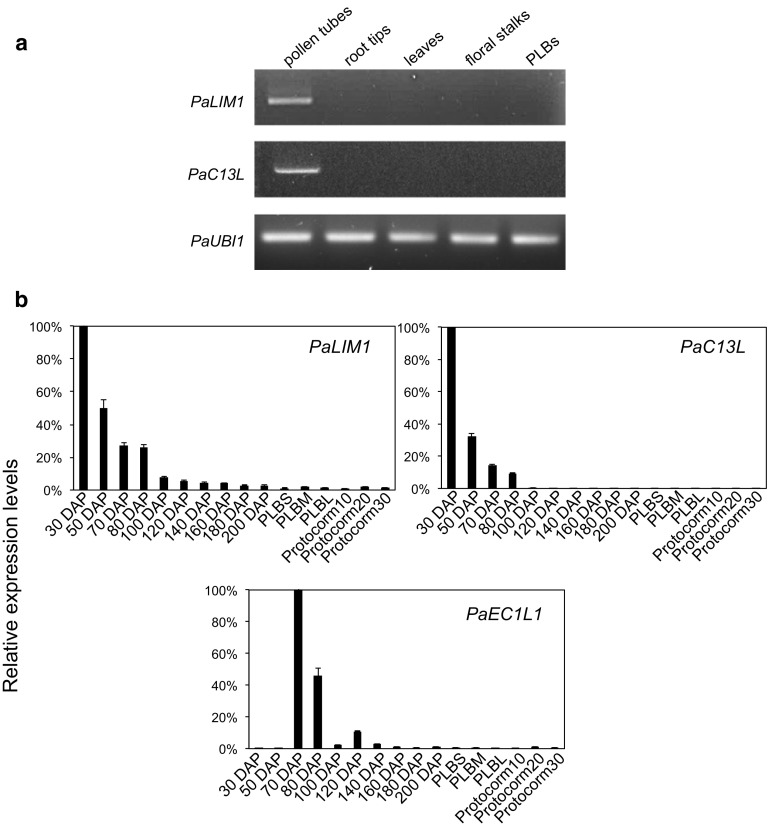
**a** Semi-quantitative RT-PCR showing that *PaLIM1* and *PaC13L* are preferentially expressed in in vitro germinated pollen tubes. *PaUbi1* was used as an internal control. **b** Quantitative RT-PCR showing expression patterns of *PaLIM1*, *PaC13L*, and *PaEC1L1* in interior tissues of developing ovaries from 30 to 200 days after pollination (DAP). Small-sized PLB (PLBS), medium-sized PLB (PLBM), large-sized PLB (PLBL), 10-day-old protocorms (protocorm10), 20-day-old protocorms (protocorm20), and 30-day-old protocorms (protocorm30)

Two genes potentially involved in cellular processes related to pollen tube growth and development were isolated by reverse transcription polymerase chain reaction (RT-PCR). They are genes encoding homologs of the LIM domain-containing proteins (Papuga et al. 2010; Wang et al. 2008) and the C13-like pollen protein (Hanson et al. 1989). Consistent with their potential functions, *PaLIM1* and *PaC13L* mRNAs were preferentially expressed in in vitro germinated pollen tubes (Fig. 5a). In addition, accumulation of *PaLIM1* and *PaC13L* mRNAs was preferentially enriched in interior ovary tissues from 30 to 70 DAP during which pollen tubes were actively growing (Fig. 5b). Their accumulation levels reached a peak at 30 DAP and gradually declined as fertilization reached completion. These data confirmed pollen-specific expression of *PaLIM1* and *PaC13L* mRNAs and showed a positive correlation between their temporal expression patterns with pollen tube activities in developing ovaries.

In addition to the pollen-specific gene, a potential egg-specific gene named *EGG CELL 1 Like 1* (*EC1L1*) gene was isolated. Arabidopsis *EC1* is an egg-specific marker gene that plays an important role to activate female and male gametes by regulating exocytosis and sperm plasma membrane modifications (Sprunck et al. 2012). To assess the timing when fertilization occurred after pollination, the expression pattern of *PaEC1L1 mRNA* was monitored in developing ovaries by qRT-PCR. As shown in Fig. 5b, the accumulation of *PaEC1L1* mRNA reached a peak at 70 DAP which coincided with arrival of pollen tubes within the embryo sacs (Fig. 2). The positive correlation between expression of *PaEC1L1* and arrival of the pollen tube at the embryo sacs further supports that fertilization occurs at approximately 65–70 DAP in *P. aphrodite*.

## Discussion

The long journey orchid pollen tubes embark upon after pollination and before fertilization have long fascinated biologists. Using the established aniline blue staining protocol, in this study we showed that pollen grains do not germinate until 3 days after pollination. It is possible that vacuolization and separation of tetrads are a prerequisite for pollen grains within the pollinia to germinate (Pacini and Hesse 2002; Pandolfi and Pacini 1995). After germination, pollen tubes continued to grow and quickly filled the entire ovary cavities as ovaries developed and elongated. Despite the continuous elongation of the pollen tubes, growth of the pollen tubes did not orient toward the developing ovules until 60–65 days after pollination. Coincidently, ovules reached maturity, marked by expression of the egg-specific marker *PaEC1L1*, at approximately the same time (Fig. 5b). It is likely that signals produced from the matured ovules are required to guide the tip-growing pollen tube in order to complete fertilization. Indeed, cysteine-rich proteins LUREs secreted from synergid cells in the matured ovules have been shown to be the key molecules that guide pollen tubes (Okuda et al. 2009).

During the course of monitoring pollen tube growth, we noticed that pollen tubes reached ovules in an acropetal manner. One of the possibilities is that ovules sequentially mature starting from the base of the ovaries (close to the pedicle) and gradually move toward the top (close to the stigma). Acropetal succession of ovule development has been documented in some plants (Bittencourt and Mariath 2002; Endress 2011). It is also possible that pollen tubes respond to matured ovules in a sequential fashion. The detailed mechanism remains to be determined. Nevertheless, to our knowledge this is the first report on sequential fertilization in the multiovulated ovary of *Phalaenopsis* orchids.

While developing the in vitro germination protocol, we found that stigmatic tissue extract is required to initiate and promote pollen germination in vitro, indicating that stigmatic tissues provide cuing molecules to signal pollen to germinate. This is not too surprising because pistil-derived molecules such as sulfinylated azadecalins (Qin et al. 2011), pistil extracellular matrix (Cheung et al. 1993, 1995), and small cysteine-rich proteins (Chae et al. 2009; Dong et al. 2005; Kim et al. 2003) have been shown to be the important stimulants for pollen germination and growth. Our established in vitro germination protocol provides a screening platform to identify substances required for pollen germination/guidance in *Phalaenopsis* orchids and maybe in other orchid species.

### **Author contribution statement**

 SCF conceived and designed the experiments. JCC performed the experiments and JCC and SCF analyzed the data. SCF wrote the paper. All authors read and approved the manuscript.
